# Atrial Fibrillation in Patients with Hypertrophic Cardiomyopathy and Cardiac Amyloidosis: From Clinical Management to Catheter Ablation Indication

**DOI:** 10.3390/jcm13020501

**Published:** 2024-01-16

**Authors:** Raffaella Mistrulli, Armando Ferrera, Melwyn Luis Muthukkattil, Allegra Battistoni, Giovanna Gallo, Emanuele Barbato, Francesco Raffaele Spera, Damiano Magrì

**Affiliations:** Clinical and Molecular Medicine Department, Sapienza University of Rome, 00185 Rome, Italy; armando.ferrera@uniroma1.it (A.F.); melwynluis.muthukkattil@uniroma1.it (M.L.M.); allegra.battistoni@uniroma1.it (A.B.); giovanna.gallo@uniroma1.it (G.G.); emanuele.barbato@uniroma1.it (E.B.); damiano.magri@uniroma1.it (D.M.)

**Keywords:** atrial fibrillation, hypertrophic cardiomyopathy, cardiac amyloidosis, catheter ablatio

## Abstract

Atrial fibrillation (AF) is the most common arrhythmia in patients affected by cardiomyopathies. Reports estimate a prevalence of 27% in patients with hypertrophic cardiomyopathy (HCM) and 40% in patients with cardiac amyloidosis (CA). The presence of AF typically results in progressive functional decline, an increased frequency of hospitalizations for heart failure, and a higher thromboembolic risk. Medical management using mainly beta-blockers or amiodarone has produced variable outcomes and a high rate of recurrence. Catheter ablation reduces symptom burden and complications despite a moderate rate of recurrence. Recent evidence suggests that an early rhythm control strategy may lead to more favorable short- and long-term outcomes. In this review, we summarize contemporary data on the management of AF in patients with cardiomyopathy (HCM and CA) with particular reference to the timing and outcomes of ablation procedures.

## 1. Introduction: Hypertrophic Cardiomyopathy and Atrial Fibrillation

HCM is the most common inherited cardiomyopathy and is caused by mutations in one of several sarcomere genes, with it being transmitted through an autosomal dominant pattern characterized by variable penetrance [1]. The most frequently involved genes are myosin heavy chain (MYH7) and myosin-binding protein C (MYBPC3). It is defined by the presence of left ventricular wall hypertrophy ≥15 mm or ≥13 mm in family members of probands. The distribution of hypertrophy generally involves the basal septum and anterior wall; however, any ventricular region may be involved.

Although AF is the most common sustained arrhythmia in both heredetary HCM and the general population, its prevalence in patients with HCM is four to six times higher than in the general population, when matched for age. Several studies have reported an overall prevalence of 20–30% and an annual incidence of 2–4% [2,3]

**Figure 1:**
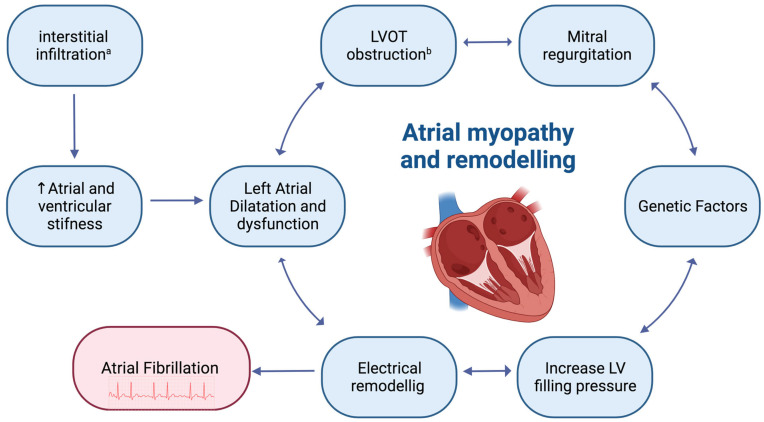
Pathophysiology of atrial fibrillation in hypertrophic cardiomyopathy and cardiac amyloidosis. ^a^ in cardiac amyloidosis; ^b^ in hypertrophic cardiomyopathy. Created with biorender.com.

Genetic factors, structural abnormalities, and electrophysiological abnormalities are probably the main factors involved in the development of AF in HCM patients [2]. Figure 1 shows the main mechanism underlying the onset of AF in HCM patients.

Accordingly, several clinical, electrocardiographic (ECG) and echocardiographic characteristics may predict the onset of AF in patients with HCM. Indeed, age, New York Heart Association functional class (NYHA III/IV), and left atrium size, with emphasis on volume rather than diameter (LAVI > 34 mL/m^2^), are better independent predictors of AF development [4,5]. LVOT obstruction in HCM is associated with worse outcomes but it appears to be an unreliable predictor [6].

The development of AF in patients with HCM is associated with a deterioration in quality of life due to a reduction in functional capacity as well as higher rates of symptomatic heart failure (HF), thromboembolism, and cardiovascular mortality, the latter being mainly related to HF progression [7]. In contrast, no association was shown between AF and sudden death. Of note, no difference in outcome has been observed with respect to the number of AF episodes in such category [8].

## 2. Pharmacological Management of AF in HCM

All patients with HCM who experience episodes of AF lasting more than 24 h should receive long-term anticoagulation to reduce the thromboembolic risk [3]. According to current recommendations, anticoagulation is also recommended for patients with HCM who experience episodes of AF lasting 5 min up to 24 h (class IIa recommendation, level C of evidence). The need for long-term anticoagulation in HCM patients with AF episodes lasting less than 5 min is currently under debate, and the choice to begin anticoagulation in these patients should be made based on each patient’s profile, by considering their bleeding risk, total AF burden, and other conventional risk factors for thromboembolism [3].

Moreover, the use of CHA2DS2-VASc score to calculate the thromboembolic risk in this population does not appear to be useful considering they already have a high baseline risk of stroke. The HCM-AF score is a novel score that uses LA diameter, age, age at HCM diagnosis, and presence of heart failure symptoms as predictive factors to estimate the risk to develop AF in HCM [9]. A score between 18 and 21 predicts a rate of AF of 1.0% to 2.0%/year, whereas a score ≥22 predicts a rate of AF >2.0%/year. Conversely, a score of ≤17 was associated with a negative predictive value of 97% on AF occurrence at 5 years [9].

Direct oral anticoagulants (DOACs) are the first choice as they have demonstrated a higher safety profile than vitamin K antagonists. According to Korean registry data on AF and HCM patients, the incidence rates of ischemic stroke, ICH, gastrointestinal hemorrhage, death, and composite outcome were all significantly lower in the NOAC group than in the warfarin group [10,11,12,13].

Rhythm control is usually the first choice in the treatment of AF. In the setting of new-onset AF, rhythm control can be achieved by electrical cardioversion and/or pharmacological cardioversion through amiodarone.

The choice of long-term anti-arrhythmic drug is guided by the expected duration of treatment, patient characteristics and existing comorbidities, and possible adverse effects. Sotalol is the most commonly prescribed anti-arrhythmic in young AF patients. Although sotalol is ineffective in cardioversion, long-term use is associated with lower rates of AF recurrence and better exercise tolerance [14]. In specific circumstances such as advanced age and imminent ablative procedure, amiodarone may be considered [1,7]. Disopyramide, a class I anti-arrhythmic drug that is typically used to treat LVOT obstruction due to its negative inotropic activity, helped maintain sinus rhythm in the early phases of therapy [1,3]. However, it has been shown to be poorly effective as long-term treatment [7].

**Figure 2:**
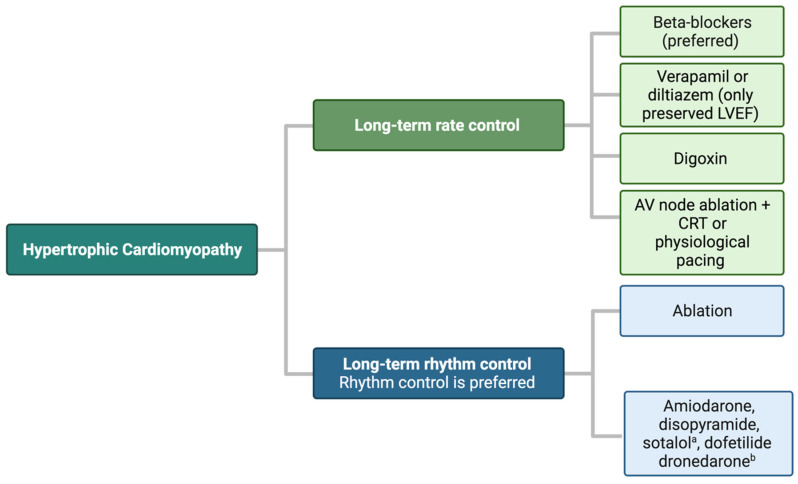
Atrial fibrillation management in hypertrophic cardiomyopathy. ^a^ Use with caution as evidence suggests that it may be associated with increased all-cause mortality. ^b^ Dronedarone is not contraindicated in LV hypertrophy but has no significant studies in HCM.

In asymptomatic patients and in those who cannot tolerate anti-arrhythmic drugs due to their adverse effects, rate control should be preferred. Recommended drugs for rate control include oral non-dihydropyridine calcium channel blockers (CCBs such as verapamil/diltiazem) or beta-blockers (metoprolol, propranolol, atenolol, nadolol), alone or in combination [1,5]. If optimal rate control cannot be achieved, digoxin may be considered alone or in combination with beta-blockers or CCBs in the long-term management of permanent AF in patients presenting with NYHA class II–IV symptoms, provided that there is no significant LVOT obstruction since the positive inotropic effect of the drug may be detrimental. Eventually, in patients with HCM for whom adequate rate control could not be achieved and for whom ablative procedures are contraindicated (i.e., significant comorbidities or advanced age), AV node ablation and pacemaker implantation may be considered. In patients with LVEF < 50%, AV node ablation may be followed by implantation through conduction system pacing (CSP) or cardiac resynchronization therapy (CRT) [15] (Figure 2).

## 3. Non-Pharmacological Management of AF in HCM

In patients with symptomatic episodes of AF who do not respond to or refuse anti-arrhythmic drug therapy, the maintenance of sinus rhythm could be achieved using an invasive approach, such as catheter ablation and the surgical maze procedure [5,16,17]. 

Although several studies have analyzed the role of catheter ablation in HCM patients for drug-refractory AF [18,19,20,21,22,23], its efficacy in terms of AF recurrence reduction and in modifying the associated electrophysiological alterations that lead to recurrence is not well established. 

A high recurrence rate has been reported in these patients, especially in early persistent and long-standing persistent AF rather than in paroxysmal AF. A significant proportion of patients require more than one ablation procedure despite long-term anti-arrhythmic therapy.

Castagno et al. [21] analyzed a cohort of 116 HCM patients undergoing radiofrequency catheter ablation for symptomatic and drug-refractory AF at four high-volume centers from 2001 to 2015.

The study population included a mix of paroxysmal (37%), persistent (44%), and long-standing persistent (19%) AF patients. Most of them underwent PV isolation plus substrate modification including additional ablation lines (LA roof and mitral isthmus lines) (65%) rather than PVI alone (19%), while a minority of patients (16%) underwent the additional ablation of complex fractionated atrial electrograms. Over a median follow-up of 6 years, only 30% of patients maintained sinus rhythm after a single procedure, with recurrence occurring after a median of 10.7 months following the index procedure. Moreover, 68% of patients with recurrence underwent a repeat ablation procedure. There were no major periprocedural complications or deaths [21]. 

In another study including 65 HCM patients, freedom from atrial tachycardia or AF during a follow-up of 48.1 ± 32.5 months occurred in 60%, after 1.9 ± 1.2 ablation procedures. In these patients, macroarrhythmias and localized atrial re-entry tachycardias were often observed after AF ablation, but the subsequent ablation of these stable atrial tachycardias resulted in effective rhythm control without anti-arrhythmic drug therapy. The ablation strategy used by this group in this setting included both wide antral PV isolation using radiofrequency (RF) energy and cryoballoon ablation. Concomitant surgical ablation was performed in patients with AF undergoing mitral valve surgery or surgical myectomy [22].

In the study by Di Donna et al. including 61 HCM patients, catheter ablation was successful with freedom from arrhythmias in 67% patients over a 29-month follow-up. However, repeat procedures were required in 52% of patients, while 54% of patients were still on anti-arrhythmic therapy. The ablation strategy consisted of point-to-point PV isolation using RF and linear lesions (LA roof and posterior mitral lines), plus a cavotricuspid isthmus line. Conduction recovery of the PV or a conduction gap along the ablation lines appeared to be the predominant causes of recurrence [23].

In contrast, Santangeli et al. found that the main cause of arrhythmia recurrence was a higher presence of non-PV-triggers in patients with HCM than in the general population. An ablation strategy based on PV and posterior wall isolation had a success rate of 91% at one year but was not effective in preventing late AF recurrence (>1 year) in about 50% of patients [18]. Most late arrhythmia recurrences presented with atypical atrial flutter, which was successfully mapped and ablated in approximately two-thirds of cases. These findings are in line with the concept that the effect of concomitant diffuse atrial fibrosis due to chronic atrial stretch secondary to diastolic dysfunction, mitral regurgitation caused by SAM of the mitral valve, and underlying atrial myopathy caused by the sarcomere protein gene mutations might provide the substrate for multiple arrhythmogenic areas beyond the PVs. Hence, the additional ablation of non-PV triggers might be an important strategy in the ablation of AF in HCM. Surgical ablation during concomitant septal myectomy has also been reported, with a satisfactory mid-term arrhythmia control at 1-year and 6-year follow-up (96 ± 3.5 and 80 ± 8.1%, respectively) [24]. Another study found that a combination of septal myectomy and Cox-III, Cox-IV, or PVI procedures resulted in a 48% 5-year recurrence of AF when compared to HCM patients with AF who underwent septal myectomy alone (59.3 ± 10.0% at 1 year and 50.8 ± 10.2% at 3 years), the combination of septal myectomy with Cox-maze IV surgical ablation resulted in statistically significant freedom from AF (90.7 ± 4.0% at 1 year and 68.7 ± 7.2% at 3 years), promoting the idea that Cox-maze IV surgery further reduces AF recurrences in these patients [25,26]. Although this procedure has been associated with higher complication rates than transcatheter ablation [27], the availability of modern tools and appropriate surgical technique in high-volume centers could allow for a significant reduction in complications. 

Regarding the main predictors of arrhythmias recurrence, left atrium size emerged as the main predictor of procedural success in all the aforementioned studies [19,20,21,22]. Di donna et al. observed that younger patients with small atrial sizes and mild symptoms had the best outcomes due to lesser degrees of atrial myopathy and remodeling [23]. 

A recent meta-analysis, including 15 observational studies, confirmed that the major predictors of AF recurrence after catheter ablation were LA size, NYHA class III/IV, AF duration, non-PV triggers, and LV systolic dysfunction [22]. One explanation for the high AF recurrence rate after the first ablation procedure could probably be that the patients included in the various trials very often had a long history of AF with even larger atria [25]. This may suggest that an early and aggressive rhythm control strategy with transcatheter ablation in HCM patients, prior to progressive atrial remodeling, may be more effective in improving quality of life and reducing long-term anti-arrhythmic drug toxicity. According to these findings, clinicians should consider AF ablation earlier in the course of disease. While further investigation will be required to understand whether an early rhythm control and first-line ablation strategy is appropriate in HCM patients, efforts should be made to identify appropriate candidates in whom substantial LA remodeling has not yet occurred and who could therefore benefit the most from AF ablation (Table 1). 

## 4. Cardiac Amyloidosis and Atrial Fibrillation

Cardiac amyloidosis (CA) is a restrictive cardiomyopathy caused by the deposition of amyloid fibrils in the myocardial interstitium [28]. There are over 15 types of systemic amyloidosis [29], each caused by a different precursor protein which promotes amyloid formation and tissue deposition, with the most common ones being light-chain amyloidosis (AL) and transthyretin amyloidosis (ATTR). In AL amyloidosis, the amyloid fibrils are composed of monoclonal immunoglobulin light chains, whilst in ATTR amyloidosis, the fibrils are composed of transthyretin protein. ATTR CA can develop in the context of variant or wild-type transthyretin genes, known as ATTRv CA or ATTRwt CA, respectively [28].

AF is the most common arrhythmia in CA and is often poorly tolerated due to the impaired ventricular filling that characterizes CA [30]. The prevalence of AF is about 20% higher in patients with CA than in the age-adjusted general population [15,31,32,33]. Its prevalence in wtATTR-CA is significantly higher than in ATTRv-CA and AL-CA (44.5% vs. 14.9% vs. 12.1%, respectively). Papathanasiou et al. [34] demonstrates that about half of patients with AL-CA or ATTR-CA have concomitant AF at presentation. 

The pathophysiological relation between AF and CA is complex and involves multiple pathways. Firstly, interstitial infiltration leads to increased ventricular wall thickness, ventricular stiffness, and consequent ventricular diastolic dysfunction [35]. The atria are also affected by amyloid deposition in the form of focal, multifocal, and/or diffuse interstitial nodules that replace normal atrial interstitial tissue which subsequently act as structural substrates for AF [36].

Together, these atrial pathological alterations represent a form of atrial cardiomyopathy that function as the pathophysiological basis for the development of AF [35,36,37,38,39,40] (Figure 1). Bandera et al. [38] characterized atrial pathology in five explanted hearts with ATTR-CA, demonstrating the presence of atrial walls’ infiltration and increased atrial stiffness. They also confirmed the coexistence of amyloid nodular deposits and mild-to-moderate fibrosis in the subendocardium. The loss of normal architecture led to abnormal atrial function that can be detected already in the early stages of atrial involvement, both with echocardiography and cardiac magnetic resonance (MRI). In patients with CA, the A wave of the transmitral flow is absent or decreased, while atrial speckle-tracking analysis generally shows the progressive impairment of the reservoir and active contraction functions [40]. Cardiac MRI usually reveals dilated and dysfunctional atria, with thickened and enhanced walls [39,40]

Age, left ventricular ejection fraction, LA diameter, mean RA pressure, longer PR, and QRS duration are the main independent risk factors for AF development in these patients [33,41].

Due to impaired ventricular filling, AF is poorly tolerated in patients with CA, with it being independently associated with a poor quality of life and an increased risk of heart failure [33,42]. 

Donnellan et al., in a recent retrospective cohort study of 382 patients with ATTR-CA, have shown no difference in mortality between patients with and without AF (65% vs. 49%; *p* = 0.76), despite a Cox proportional hazards analysis having linked the maintenance of sinus rhythm and tafamidis use with improved survival [43].

## 5. Pharmacological Management of AF in CA

CA patients with AF have a notably increased risk of intracardiac thrombus, stroke, and systemic thromboembolism regardless of CHA_2_DS_2_-VASc score [42,43,44,45,46,47]. Regardless of AF, this category of patients is at high risk of stroke and systemic embolism. 

According to 2023 European Society of Cardiology (ESC) guidelines for the management of cardiomyopathies, oral anticoagulation, unless contraindicated, is recommended in all patients with CA and AF, regardless of their CHA_2_DS_2_-VASc score, in order to reduce the risk of stroke and thrombo-embolic events (class I level of evidence B) [45]. Conversely, the role of anticoagulation in CA patients on sinus rhythm remains a gray area [48].

Although no data exist from randomized controlled trials with respect to the possible advantages of new oral anticoagulants versus VKAs in CA patients with AF, recent data suggest that DOACs can be used safely and effectively in CA patients [49].

Due to the importance of atrial contraction in determining the LV filling in CA patients, [33], the rhythm control strategy is preferred over rate control, particularly in symptomatic patients [45]. Among anti-arrhythmic drugs, amiodarone appears to be well-tolerated in the CA population and is the anti-arrhythmic of choice [45]. However, from a clinical viewpoint, there is no solid evidence to confirm the superiority of a rhythm control strategy over a rate control strategy in terms of clinical outcomes [48].

**Figure 3:**
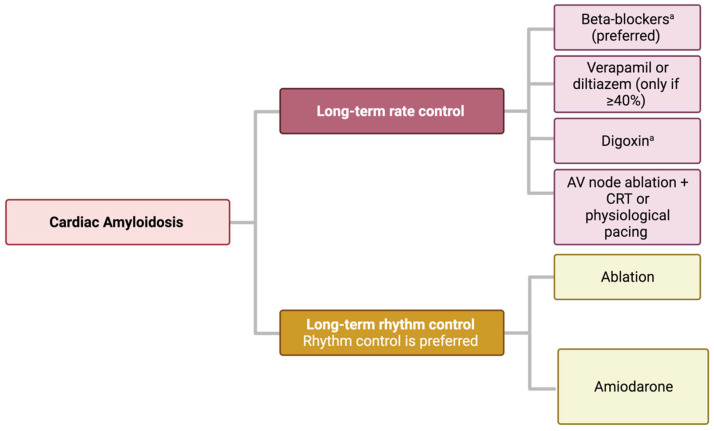
Atrial fibrillation management in cardiac amyloidosis. ^a^ Beta-blockers in low dosage and digoxin with caution.

When rhythm control fails, rate control becomes essential, even if challenging. Indeed, patients with CA often have severe diastolic dysfunction with restrictive characteristics that require compensatory tachycardia in order to maintain adequate cardiac output, which account for the poor tolerance associated with the use of beta-blockers, CCB, and digoxin [47,50]. Moreover, these drugs could precipitate hypotension in the setting of concomitant autonomic dysfunction that typically occurs in CA [51] and could aggravate or generate cardiac conduction defects [52,53,54]. Guidelines and consensus documents [28,45] recommend a low dose of beta-blockers as first-line treatment in the setting of a rate control strategy in CA. Digoxin can be used with caution as a second-line treatment, while verapamil or diltiazem should be reserved for patients with LVEF ≥40%. In patients with CA and poor ventricular rate control despite optimal medical therapy and who are not candidates for rhythm control by catheter ablation, atrioventricular node ablation with CRT or physiological pacing is an alternative [45] (Figure 3).

## 6. Non-Pharmacological Management of AF in CA

As previously mentioned, rhythm control is preferred over rate control in patients with AF and CA. Furthermore, in the absence of randomized control trials studying the safety and efficacy of catheter ablation in this setting, a pharmacological strategy is currently preferred over an invasive strategy [45]. However, numerous studies have been conducted in recent years to investigate the safety and efficacy of catheter ablation in this setting. Barbhaiya et al. [55] showed that patients with CA affected by atrial arrhythmias and conduction system disease (HV > 55 ms) who underwent catheter ablation had a high recurrence rate of tachyarrhythmias compared to age-matched, non-CA comparator AF patients (hazard ratio: 5.4; 95% confidence interval: 1.9 to 35.5; *p* = 0.007). In a single-center study by Tan et al. [56] consisting of 26 patients with CA and atrial arrhythmias (AAs) (AFL, AF, AT), 13 patients underwent catheter ablation and 13 underwent atrioventricular nodal ablation. Five patients in the ablation group underwent only pulmonary vein isolation. The overall 1-year and 3-year recurrence-free survival rates were 75% in the catheter ablation group and 60% in the atrioventricular nodal ablation. Complication rates were not reported. Donnellan et al. [57] showed the outcomes of 72 patients with ATTR CA and AF in a retrospective observational cohort study. In 24 of these 72 patients, catheter ablation was performed, while the remaining 48 were treated with medical management (12 patients of those 48 with AADs). In the ablation group, radiofrequency ablation was performed in all cases. Despite no difference in clinical and echocardiographic parameters, the recurrence rate of AF was 58% after a mean FU of 39 ± 26 months. Compared with controls, the ablation group had lower rates of death (29% vs. 75%; *p* = 0.01) and of hospitalizations for heart failure or arrhythmias (1.7 ± 2.4 vs. 4 ± 3.5; *p* = 0.005). The authors concluded that AF ablation is safe and is associated with reduced mortality in ATTR-CA. Another study by Alhassan et al. [58] compared catheter ablation for AF in patients with CA compared to matched patients with dilated cardiomyopathy. The authors reported no differences in the rates of complications (14.3% vs. 10.5%, *p* = 0.60), and the safety outcomes were similar to a propensity score-matched cohort of dilated cardiomyopathy. The latest study by Ullah [59] using global health data from 148,133 patients with AF and heart failure who underwent AF ablation identifies a cohort of 616 patients (293 with CA and 323 without CA). The authors stated that AF ablation is associated with significantly higher in-hospital all-cause mortality (adjusted OR 9.03, 95% CI 1.12–72.70). In conclusion, considering the small sample of these studies, the important limitations due to the design of a retrospective study, and the contrasting results, the utility and role of catheter ablation for AF remains a matter of debate. Considering the lack of randomized data, more studies are needed to determine if it represents an effective rhythm control strategy.

## 7. Conclusions

AF is the most common arrhythmia in HCM and CA patients, with it being poorly tolerated. It is linked to several processes, including genetic factors, LA structure, and electrical remodeling. AF in these patients is associated with worsening heart failure, functional decline, increased risk of thromboembolism, and increased mortality. Anti-arrhythmic drugs are ineffective in maintaining sinus rhythm while conferring an increased risk of extracardiac adverse effects. Transcatheter ablation may therefore represent an increasingly suitable approach, especially if performed in the early stages of the disease. However, large multicenter studies are required to evaluate the most effective ablation strategy and the associated long-term outcomes.

## Figures and Tables

**Table 1:** Published studies of atrial fibrillation ablation in patients with hypertrophic cardiomyopathy. Values are indicated as total number (*n*), percentage (%), or mean ± SD. AF, atrial fibrillation; AT, atrial tachycardia; CFAE, complex fractionated atrial electrograms; CTI, cavotricuspid isthmus; FU, follow-up; PVI, pulmonary vein isolation; SA, surgical ablation; and SVC, superior vena cava.

Author	Study Design	No. of Patients	Persistent AF %	Ablation Procedure	Occurrence of AT, %	Freedom of AF/AT, %	No. of Procedures	FU Duration, y
Di Donna et al. (2010) [23]	Retrospective multicenter	61	43	PVI, roof, mitral line, CTI (in 15 patients)	15	67	1.5	2.4 ± 1.3
Santangeli et al. (2013) [18]	Prospective multicenter	43	72	PVI, box lesion, SVC isolation, CFAE, non-PV trigger	37	94	1.6 ± 0.7	1.3 (0.7–1.6)
Derejko et al. (2013) [19]	Prospective observational	30	53	PVI, CTI, mitral line, roof, CFAE	n.a	53	1.4	1.9 ± 1.2
Bassiouny et al. (2015) [20]	Retrospective single-center	CA 79 (54), SA 68 (46)	42	PVI, mitral line, roof, CTI, Cox-Maze	8	46	1.2	2.9 (1.2–5)
Dinshaw et al. (2020) [22]	Retrospective single-center	65	79	PVI, CFAE, AT as appropriate	38	60	1.9 ± 1.2	4.0 ± 2.7
Castagno et al. (2021) [21]	Prospective multicenter	116	63	PVI, LA LINES, CFAE	70	61	1.6	6.1 ± 3.5

## Data Availability

Not applicable.
